# Genetic variation of aldolase from Korean isolates of *Plasmodium vivax* and its usefulness in serodiagnosis

**DOI:** 10.1186/1475-2875-11-159

**Published:** 2012-05-08

**Authors:** Jung-Yeon Kim, Hyung-Hwan Kim, Hyun-ll Shin, Youngjoo Sohn, Hyuck Kim, Sang-Wook Lee, Won-Ja Lee, Hyeong-Woo Lee

**Affiliations:** 1 Division of Malaria and Parasitic Diseases, National Institute of Health, Korea Centers for Disease Control and Prevention, Cheongwon-gun, 363-951, Republic of Korea; 2 Vascular Medicine Research Unit, Brigham and Women’s Hospital, Harvard Medical School, Cambridge, MA, 02139, USA; 3 Department of Anatomy, College of Oriental Medicine, Institute of Oriental Medicine, Kyung Hee University, Hoegi-dong, Dongdaemun-gu, Seoul, 130-701, Republic of Korea; 4 International Research Center for Bioscience and Biotechnology, Jungwon University, Goesan, 367-805, Republic of Korea; 5 Department of Pathology, University of Florida, J-566, 1275 Center Drive, Gainesville, FL, 32610, USA

## Abstract

**Background:**

The malaria aldolase is widely used as rapid diagnostic test (RDT), but the efficacy in aspect of its serological effectiveness in diagnosis is not known. The genetic variation of Korean isolates was analysed and recombinant aldolase was evaluated as a serological antigen in *Plasmodium vivax* malaria.

**Methods:**

Genomic DNA was purified and the aldolase gene of *P. vivax* from 25 patients’ blood samples was amplified. The samples came from 5 epidemic areas; Bucheon-si, Gimpo-si, Paju-si of Gyeonggido, Gangwha-gun of Incheon metropolitan city, and Cheorwon of Gangwon-do, South Korea. The antigenicity of the recombinant aldolase was tested by western blot and enzyme-linked immunosorbent assay (ELISA).

**Results:**

Sequence analysis of 25 Korean isolates of *P. vivax* showed that the open reading frame (ORF) of 1,110 nucleotides encoded a deduced protein of 369 amino acids (aa). This ORF showed 100% homology with the *P. vivax* Sal I strain (XM_00165894) and *P. vivax* WDK strain (AF247063), 87.4% homology with *Plasmodium falciparum* (AF179421), 90.6% homology with *Plasmodium chabaudi* (AF247060), 89.5% homology with *Plasmodium vinckei* (AF247061), and 96.7% homology with *Plasmodium knowlesi*. A single nucleotide polymorphism (SNP) at nucleotide 180 (G to A, n = 5) was also observed in the isolates. The expressed recombinant protein had a molecular weight of approximately 31 kDa (monomeric form) and 62 kDa (dimeric form) as analysed by sodium dodecyl sulfate-polyacrylamide gel electrophoresis (SDS-PAGE) analysis. Among 109 *P. vivax* patients, 32 (29.4%) had positive in an enzyme-linked absorbance assay (ELISA). This result showed significant correlation between ELISA and an indirect fluorescent antibody test (IFAT) (*P* < 0.0001).

**Conclusions:**

The aldolase gene from Korean isolates of *P. vivax* showed one SNP at nucleotide position 180; this SNP mutant was discovered in only the western part of Han River, and included the regions of Ganghwa, Gimpo, and Bucheon. Based on the results, the relationship between antibody production against aldolase and the pattern of disease onset should be more investigated before using aldolase for serodiagnosis.

## Background

Microscopic examination of the *Plasmodium* species is regarded as the gold standard method for malaria diagnosis. Despite the simplicity and low cost, it is not always available [1]. Over the last ten years, the development of alternative diagnostic tests for malaria, such as rapid diagnostic tests (RDTs), has made it possible to extend biological diagnosis to remote areas with few resources. These lateral-flow immunochromatographic tests detect specific antigens produced by malaria parasites and are rapid and simple to carry out without electricity, specific equipment or intensive training [2-4].

To detect *Plasmodium*, monoclonal antibodies against lactate dehydrogenase (pLDH), histidine-rich protein-2 (HRP-2) and aldolase are widely used [5,6]. The genetic diversity of HRPII is known to partly influence the sensitivity of RDT [7]. It is known that the genetic variation of aldolase genes is very low [8,9], but it is not clear if this is true in the Korean isolates of *Plasmodium vivax*. Aldolase is a key enzyme that helps convert glucose into energy in malaria parasites. Meier et al found that two type of aldolase, aldo-1 and aldo-2, from *Plasmodium berghei*. aldo-1 of *P. berghei* was found to be virtually identical to the aldolase gene of *Plasmodium falciparum*, whereas aldo-2 had 13% sequence diversity. In addition, aldo-1 was detected in the sporozoite stage while aldo-2 was detected in the asexual stages of malaria parasites with specific antibody probes [10].

In this study, it was investigated that genetic variation of aldolase genes which were isolated from 25 patients who lived in 5 geographically different epidemic areas in South Korea and evaluated the recombinant protein as a serodiagnostic tool.

## Methods

### Blood sample collection

Patients with clinically suspected malaria attending the Public Health Centres in Gangwha-gun, Gimpo-si, Bucheon-si, Paju-si of Gyeonggi-do and Cheorwon-gun of Gangwon-do, South Korea in 2011, were examined for malaria parasites. Approximately 3 ml of blood was collected from each symptomatic patient. Thin and thick blood smears were prepared for microscopic examination (magnification 7 × 100). Blood samples were transported to the Korean National Institute of Health (KNIH), where sera were separated and stored at −20°C for future antibody analysis. Informed consent was obtained from all patients, and all samples were collected under human use protocols that have been reviewed and approved by the Human Ethics Committee of the National Institute of Health (Osong, Korea).

### Amplification of the aldolase gene

For the purpose of expression of the aldolase gene, genomic DNA was extracted from the whole blood of a malaria patient using a QIAamp Blood Kit (Qiagen, Hilden, Germany). PCRs were performed using AccuPower PCR Premix (Bioneer, Taejeon, Korea), 50 ng of purified genomic DNA, 40 pmoles each of forward (AF1; 5′- GCC ACT GGA TCC GAA TAT AAA AAC GCC CCC-3′) and reverse primers (AR1; 5′- ATA GAC GTA CTT CTT TTC GTA AAG GGA TGC-3′) and the total volume was adjusted to 20 ml with distilled water. The thermocycler conditions were as follows: denaturation at 94°C for 5 min, 35 cycles of 1 min at 94°C, 1 min at 55°C and 2 min at 72°C, and finally incubation at 72°C for 5 min. All of the PCR products were analysed on a 1.2% agarose gel, confirmed under a UV transilluminator and purified with a NucleoSpin Extract Kit (Macherey-Nagel, Duren, Germany). The purified PCR products were ligated with pCR2.1 cloning vector (Invitrogen, Carlsbad, CA, USA) and then transformed into *Escherichia coli* INVα F’ according to the procedures of Invitrogen.

### DNA sequencing and analysis

The PCR product inserted into *E. coli* was selected for on ampicillin and X-gal containing medium. To confirm the transformants, gel electrophoresis was performed with *Eco*RI digestion products after preparation of the plasmid with a Qiagen plasmid isolation kit, according to the protocol supplied by the manufacturer. The aldolase gene sequence was determined using ABI PRISM dye terminator cycle sequencing ready reaction kit FS (Perkin Elmer, Cambridge, MA, USA) according to the supplied manual. M13 reverse and M13 forward (−20) primers were used in sequencing. Nucleotide and deduced amino acid sequences were analysed using EditSeq and Clustal in the Megalign program, a multiple alignment program in the DNASTAR package (DNASTAR, Madison, WI, USA). The internet-based BLAST search program of the National Centre for Biotechnology Information (NCBI) was used to search protein databases. The gene sequences of aldolase from the Korean isolates were deposited in GenBank (Accession No. JN181172-JN181196).

### Construction of the aldolase expression vector

For the expression of aldolase gene of Pv Kor12 type strain in *E. coli*, the gene fragment was amplified from the DNA of blood samples that were confirmed to be infected with *P. vivax* as described above, and which have the *Sma*I and *Sal*I sites on their 5′ ends. Amplified PCR products were digested with *Sma*I and *Sal*I, purified with a Qiagen Gel Extraction Kit after being run on an agarose gel and were then integrated into the *Sma*I and *Sal*I cleavage sites of the pQE80 expression vector (Qiagen). The resulting plasmid was subsequently used for the expression of the aldolase fusion protein in *E. coli* DH5a [11]. Transformants were confirmed both by gel electrophoresis of plasmid DNA after restriction enzyme digestion with *Sma*I and *Sal*I.

### Expression and purification of recombinant aldolase

Expression of the recombinant protein was induced in *E. coli* with isopropyl-1-thio-β-D-galactopyranoside (IPTG) [12]. 1 mM IPTG was added to cultures of *E. coli* DH5α (pVKor12) grown to a logarithmic phase in liquid LB containing 100 μg/ml ampicillin and 25 μg/ml kanamycin to induce expression of the target protein and purification of the aldolase fusion protein was carried out using immobilized metal ion affinity chromatography [13]. The purification was done under native conditions according to the supplier’s protocol (Qiagen). Proteins were analyzed on sodium dodecyl sulfate-polyacrylamide gel electrophoresis (SDS-PAGE) in each purification step.

### Western blot analysis

Recombinant aldolase fusion protein was separated on a 12% SDS-PAGE gel and was then transferred to a nitrocellulose membrane. After the transfer, the membrane was cut into strips and blocked for non-specific binding with 5% skim milk for 12 hrs at 4°C. The membrane was then washed in PBS with 0.15% Tween 20 for 3 × 10 min. The strips were allowed to react with sera of malaria patients or of uninfected people (diluted 1:100, vol/vol) for 4 hrs and then washed using the procedure described above. The membrane was then incubated with diluted peroxidase-conjugated goat anti-human IgG secondary antibody (1:1,000, v/v) (Sigma) for 3 hrs at room temperature. For colour development, a solution containing 0.2% diaminobenzidine and 0.02% H_2_O_2_/PBS was applied to each well [14,15].

### Enzyme-linked immunosorbent assay

Sera from patients infected with *P. vivax* were analysed for the presence of antigen-specific antibody using 96-well plates coated with 0.5 mg/ml purified recombinant protein that had been expressed in *E. coli* and dissolved in phosphate-buffered saline (PBS, pH 7.4) overnight at 4°C. Malaria patient serum was diluted 1:100 (v/v) in blocking buffer (0.25% PBS-Tween 20 with 1% bovine serum albumin, pH 7.4) and incubated for 1 hr, followed by incubation with peroxidase-conjugated goat anti-human IgG secondary antibody at a 1:1,000 dilution (v/v, Sigma). Optical density was measured with a spectrophotometer at 405 nm (Molecular Devices, Sunnyvale, CA) [15]. It was regarded as positive sera showed over the cut off value which obtained by mean ± 2 X standard deviation (SD) of negative controls.

### Indirect fluorescent antibody test

To test for antibodies against malaria, indirect fluorescent antibody test (IFAT) was performed using the whole blood antigen for *P. vivax*[16-18]. Briefly, 10 ml of malaria parasite-infected blood was collected by venopuncture from *P. vivax* patients. After plasma removal, the blood cells were suspended in phosphate buffered saline (PBS, pH 7.2) and centrifuged for 5 min at 2,500 rpm. The supernatant was discarded and the cells were resuspended in fresh PBS. This wash step was repeated three more times, and then an appropriate amount of PBS was added to maintain the parasitaemia at no less than 1%. Cells were added to each well contained in Teflon coated slides. After 12 hrs drying at room temperature, the slides were stored at −70°C. In order to determine the antibody titres against *P. vivax* for each patient, the antigen slides were fixed in pre-cooled acetone (−20°C) for 10 min, washed with PBS, and then 20 μl of diluted sera, 1:32 to 1:8,192 (vol/vol), was added to each well. Positive and negative controls were also spotted onto each slide and then the slide was incubated in a humidified chamber for 30 min at 37°C. The reactions on the slides were quenched by washing the reacted sera with PBS for 6 min, and then the slides were dried at room temperature. Diluted FITC conjugated anti-human IgG (Sigma, 1:32 vol/vol in PBS) was added to each well and then the slides were incubated and washed using the same method described above. Several drops of buffered glycerol were added to the samples, and then coverslips were applied. The slides were examined under the 40x objective of a fluorescence microscope.

### Data analysis

The relationship between ELISA and IFAT positive rate was analyzed by one-way ANOVA. Data analyses were performed using GraphPad (GraphPad Software, Inc., La Jolla, CA, USA).

## Results

### Blood collection and sequence variation of aldolase from Korean isolates of *Plasmodium vivax*

**Figure 1:**
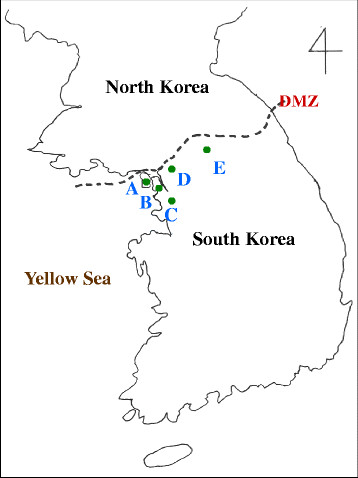
**Studied areas. A;** Ganghwa-gun, **B;** Gimpo-si, **C;** Bucheon-si, **D;** Paju-si, **E;** Cheorwon-gun. DMZ; demilitarized zone.

**Figure 2:**
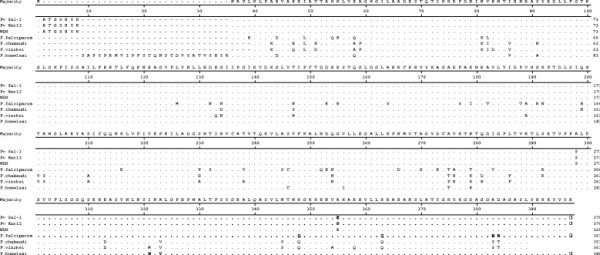
**Multiple amino acid sequence alignment.** The deduced amino acid sequence of Pv Kor12 type strain in Korean isolates of *P. vivax* was aligned with those from other *Plasmodium* species. Computer analysis was performed using the multiple sequences alignment of MegAlign. All amino acid sequences were obtained from GenBank BLAST (http://WWW.ncbi.nlm.nih.gov). *P. vinckei* (Accession; AF247061), *P. chabaudi* (AF247060), WDK; *P. vivax* WDK (AF247063), Pv Kor12; type strain from *P. vivax* Korean isolate, Pv Sal-1; Sal-1 strain of *P. vivax* (XM_00165894), *P. falciparum* (AF179421), and *P. knowlesi* (XM_002260389).

**Figure 3:**
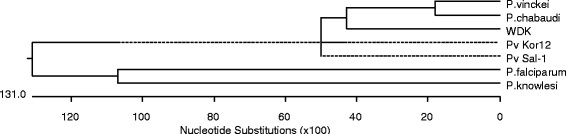
**Phylogenetic relationships between the aldolase genes of several strains of *Plasmodium*.** Computer analysis was performed using the multiple sequences alignment of MegAlign. All amino acid sequences were obtained from GenBank BLAST (http://WWW.ncbi.nlm.nih.gov). *P. vinckei* (Accession; AF247061), *P. chabaudi* (AF247060), WDK; *P. vivax* WDK (AF247063), Pv Kor12; Type strain of from *P. vivax* Korean isolate, Pv Sal-1; Sal-1 strain of *P. vivax* (XM_00165894), *P. falciparum* (AF179421), and *P. knowlesi* (XM_002260389).

The geographical locations of study areas were expressed in Figure 1. Gangwha-gun, Gimpo-si, and Bucheon-si are located along the western part of Han River, which is the largest river that passes through Seoul, South Korea. Paju-si and Cheorwon-gun are located along the eastern part of Han River. Five blood samples infected with indigenous *P. vivax* were collected from each city in 2011. Among 25 Korean isolates of aldolase, 12 isolates had the same DNA and amino acid sequences as *P. vivax* Sal-1 (XM_001615894) and these were designated as strain type Pv Kor12. The aldolase from these isolates had 1110 nucleotides and 386 amino acids. When the amino acid of Pv Kor12 was compared with several *Plasmodium* species, Pv Kor12 showed 100% homology with *P. vivax* WDK (AF247063, 1110 bp), 87.4% homology with *P. falciparum* (AF179421, 1089 bp), 90.6% homology with *P. chabaudi* (AF247060, 1054 bp), 89.5% homology with *P. vinckei* (AF247061, 1054 bp), and 96.7% homology with *P. knowlesi* (XM_002260389, 1170 bp) (Figures 2 and 3).

**Figure 4:**
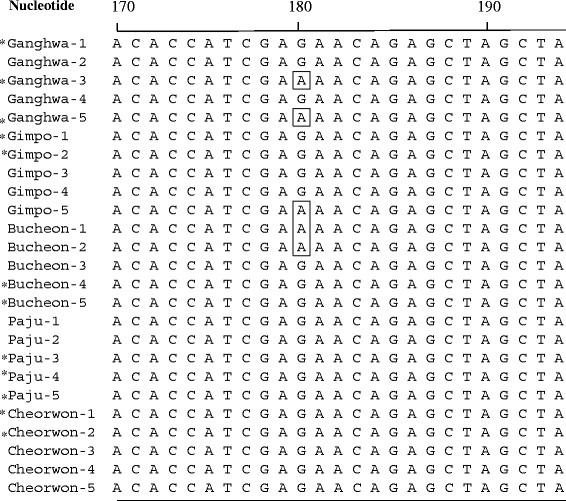
**Comparing the single nucleotide polymorphism (SNP) of the aldolase gene between 25* Plasdodium vivax *Korean isolates.** *; represents the isolate belong to PvKor12. All amino acid sequences were deposited in GenBank BLAST (http://WWW.ncbi.nlm.nih.gov, Accession No. JN181172-JN181196).

The aldolase gene was amplified from the genomic DNA of 25 Korean isolates. Amplification of the aldolase gene yielded a product of approximately 1,100 base pairs. After purification, the DNA fragment was ligated into the pCR 2.1 cloning vector (3.9 kb). The plasmid containing the PCR product was named pValdol (5.0 kb) and was used for DNA sequence analysis. Based on DNA sequencing, the cloned aldolase gene was shown to be 1,110 bp and consisted of 386 amino acids that were deduced by DNASIS. One synonymous single nucleotide polymorphism (SNP) at base pair 180 (n = 5), from G to A, was observed based on literatures [6,8]. Two of SNP were from Gangwha-gun, one from Gimpo-si, two from Bucheon-si, and none were from Paju-si or Cheorwon-gun (Figure 4). Rest of them (n = 8) has one or two SNPs, but those locations did not show any common feature. These should be cleared through the analysis of the more DNA samples.

### Expression of aldolase in *E. coli*

**Figure 5:**
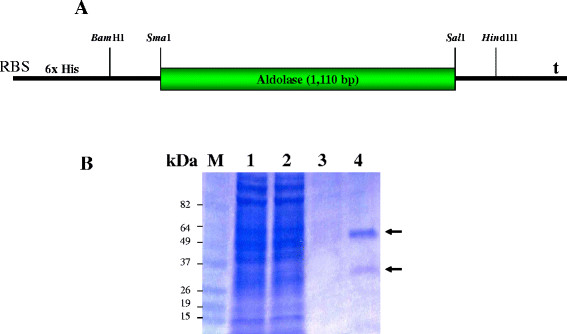
**Gene structure for the expression of Pv aldolase (A). Patterns of purified aldolase by SDS-PAGE under native conditions using Ni-NTA agarose resin (B).** Lane 1: Crude cell lysate, Lane 2: Flow-through, Lane 3: 20 mM imidazole wash, Lane 4: 250 mM imidazole elution, Lane M: Protein size marker. RBS: ribosome binding site, 6xHis: 6x Histidine tag sequence, t: stop codon.

The resultant plasmid pVKor12 contained the aldolase gene fused to a (His)_6_-tag (Figure 5A). The recombinant plasmid pVKor12 was then transferred into *E. coli* DH5α. As analysed on SDS-PAGE followed by Coomasie blue staining, two forms of recombinant aldolase protein were observed under native purification conditions, a dimeric form (62 kDa) and a monomeric form (31 kDa) (Figure 5B).

### Antigenicity of the aldolase recombinant protein

**Figure 6:**
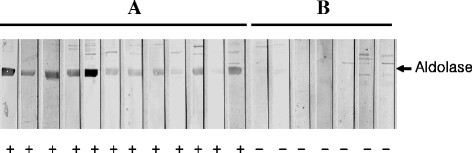
**Western blot analysis of aldolase recombinant protein. A:** Malaria patients, **B:** Healthy individuals.

**Figure 7:**
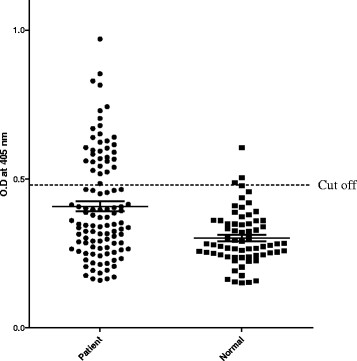
**Scattergram of absorbances measured by ELISA using aldolase recombinant protein.** Sera from healthy individuals and malaria patients infected with *P. vivax* were used. Cut off showed the mean ± 2 X SD of negative controls.

**Figure 8:**
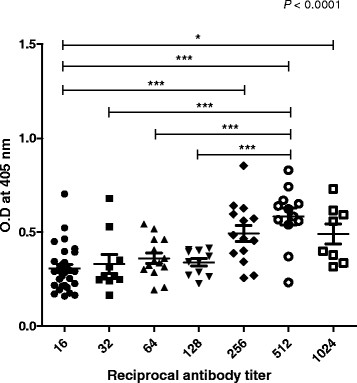
**Comparison the values measured by IFAT with whole blood antigen and the value obtained by ELISA with aldolase recombinant protein.** Significance was evaluated by the one-way ANOVA. *; *P* < 0.05, ***; *P* < 0.0001.

To determine the antigenicity of the aldolase recombinant protein by western blot, ELISA, and IFAT, sera of malaria patients which had been reserved in KNIH collected between 2009 and 2010. Negative sera were collected from volunteers of stuffs in KNIH. Sera of twelve malaria patients showed positive reaction by western blot while the sera from the normal control group (n = 7), who had never been exposed to malaria, tested negative (Figure 6). After the number of malaria patients was increased, the antigenicity of recombinant aldolase was evaluated by ELISA. Thirty-two of 109 sera (29.4%) from malaria patients, as confirmed by microscopic analysis, reacted with the aldolase recombinant antigen. In addition, 3 of 67 normal persons (4.5%) reacted with the aldolase recombinant antigen (Figure 7). The reaction intensity of the recombinant aldolase antigen with patient sera increased significantly depends on the positive reactions of each serum dilution in IFAT (Figure 8, *P* < 0.0001). The intensity of ELISA reached a maximum level at a 1:512 serum dilution of IFAT.

## Discussion

Aldolase is an important enzyme involved in the glycolysis pathway of malaria parasites. Higher vertebrates usually has three kind of tissue-specific aldolase isoenzymes [19], however, *P. falciparum* and *P. vivax* posses only one aldolase isoenzyme [20,21], similar to *Trypanosoma brucei*[22]. The aldolase enzymes of *P. falciparum* and *P. vivax* are both 369 amino acids long and their nucleotide and amino acid sequences are relatively conserved than other genes’ variation [21]. However, genetic variation of the aldolase gene of Korean isolates is not analysed systematically. Therefore, it is important to understand the genetic variation of the aldolase gene in Korean isolates.

For the 110 *P. vivax* aldolase gene sequences from Madagascar isolates, only two synonymous changes were observed. A SNP at nucleotide 510 (TCC to TCA) was found in one isolate from the Tsiroanomandidy area, whereas an SNP at nucleotide 651 TTA to TTG) was observed in 43 isolates from the other regions [23]. Recently, two synonymous SNPs were found among 84 Korean isolates, four cases has changed nucleotide from G to A at base pair 180, two cases has the same nucleotide change at base pair 645 [6]. However, only one kind of synonymous SNP was found at base pair 180 (n = 5) in 25 Korean isolates. SNP at base pair 645 has not been found in this study. It may caused by sample number used in this study. Together with Cho *et al*[6] and ours data, SNP at base pair 180 is dominant mutation than SNP at base pair 645 in South Korea. Interestingly, mutant strains were isolated from the western part of Han River, which has an approximate length of 514 Km. Within the study area, the river is more than 1 Km wide. Therefore, the SNP found in the aldolase gene sequence may be related to the geographical isolation caused by the river. The flight distance a night of *Anopheles sinensis*, the main mosquito vector in Korea, is mainly limited to 1 Km [24], so the river is big enough to prevent mosquitoes crossing the river directly.

*Plasmodium vivax* has presumably been prevalent in Korea for a long time. However, as a result of a national malaria eradication programme and with help from the WHO, the incidence of vivax malaria has rapidly decreased [25,26]. After the latest report of two malaria patients in 1985 [27], there were no additional reported cases until one case was reported in 1993 [28] and two indigenous cases were reported in 1994 [29]. Malaria cases then rapidly increased until around 2000 [30,31]. After that, the reported malaria cases were reduced for several years due to efforts to limit the incidence of malaria. However, malaria was not thoroughly eradicated in the Korean peninsula because 2–3% of patients experience failed drug treatment every year, and many travellers and workers come from malaria-prevalent areas, including North Korea [32]. For these reasons, serological diagnostic tools are needed to support both traditional microscopic diagnosis and seroepidemiological studies for estimating the prevalence of malaria in Korea. Currently, IFAT is used as the gold standard method due to its high sensitivity. However, the sensitivity might be affected by the ability of examiners. Therefore, a new technique is needed for serodiagnosis. Several recombinant proteins cloned from Korean isolates of *P. vivax* were tested for use as the antigen for serodiagnosis, including circumsporozoite protein (CSP) –subtypes Pv210 [33] and Pv247 [34], merozoite surface protein (MSP) [35], as well as CSP and MSP chimera proteins [36,37]. None of these antigens were sufficient to replace the IFAT method, because the sensitivity was less than IFAT. Therefore, it was decided to focus on the enzyme aldolase. Monoclonal antibodies against aldolase were used in several RDTs and showed a relatively high sensitivity in detecting malaria parasites, including Korean isolates [6].

However, the sensitivity of aldolase was 29.4% (32/109), even though it was cloned from a type of Korean *P. vivax* strain (Pv Kor12, Figure 2). There may be many reasons and explanations for the low seroconversion rate detected by ELISA using recombinant aldolase antigen (Figure 7). The same situation happened for the circumsporozoite protein (CSP) of *P. vivax*. The basic reason for the low seroconversion rate for CSP was linked to a low exposure of this developmental stage to dendritic cells. When the seroconversion rate of Korean malaria patients against CSP antigen was analysed, it was shown to be less than 30%. Therefore, a hypothesis was proposed to explain this observation. First, this may be explained by the antigenicity of CSP or the exposure time of contact between DC and CSP before entering the liver cells. Second, it may be explained by the relative short half-life time of CSP antibody in relation to onset time of illness after a long incubation period [30,33]. This portion was 70% and this matches well the portion of antibody positive rate of CSP (30%) and the percentage of malaria patients who had a short incubation period (30%). By analogy, this may explain the low seroconversion rate of aldolase. This hypothesis should be tested using blood samples from long and short incubation period patients, but it is not easy to identify patients with long and short incubation periods. Recently, two types of aldolase from *P. berghei* were identified. Aldo-1 was identical to the *aldolase* gene of *P. falciparum* and aldo-1 was detected in the sporozoite stage, while aldo-2 was detected in the asexual stages of malaria parasites [10]. Therefore, it is conceivable that the currently known aldolase of *P. vivax* also belongs to the sporozoite stage. This may explain why recombinant aldolase showed a low seroconversion in this study. Additionally, if the blood stage-specific isoform of aldolase was obtained, it may have much higher seroconversion rate in ELISA.

## Conclusions

The aldolase gene from *P. vivax* Korea isolates has an SNP at 180 (G to A) and, interestingly, the mutant strains were only found along the western part of the Han River. New information of geographic mapping of aldolase at the national or regional scale would provide a valuable aid to developing and updating national anti-malarial policy guidelines in Korea. Additionally, more information is needed before using aldolase as a serological diagnostic antigen.

## Competing interests

The authors declare that they have no competing interests.

## Authors’ contributions

JYK, HHK, WJL and HWL conceived and designed the study and contributed to the execution of the research. HWL wrote the manuscript. HHK, SY, and HK contributed statistical analysis. YJK, HIS, and WJL collected the blood samples in the field. YJK, SWL, and HIS performed preparing the DNA samples for DNA sequencing, IFAT and ELISA. All authors have read and approved the final manuscript.
